# Systemic high-mobility group box 1 administration suppresses skin inflammation by inducing an accumulation of PDGFRα^+^ mesenchymal cells from bone marrow

**DOI:** 10.1038/srep11008

**Published:** 2015-06-05

**Authors:** Eriko Aikawa, Ryo Fujita, Yasushi Kikuchi, Yasufumi Kaneda, Katsuto Tamai

**Affiliations:** 1 Department of Stem Cell Therapy Science, Graduate School of Medicine, Osaka University, Japan; 2 Division of Gene Therapy Science, Graduate School of Medicine, Osaka University, Japan

## Abstract

High-mobility group box 1 (HMGB1) mobilizes platelet-derived growth factor receptor alpha-positive (PDGFRα^+^) mesenchymal cells from bone marrow (BM) into circulation. However, whether HMGB1-induced endogenous PDGFRα^+^ mesenchymal cells stimulate skin regeneration has been unclear. Here, we investigated the functions of the HMGB1/BM-PDGFRα^+^ mesenchymal cell axis in the regeneration of mouse skin grafts. We found that intravenous HMGB1 administration induced an accumulation of endogenous BM-PDGFRα^+^ mesenchymal cells followed by significant inflammatory suppression in the grafts. In contrast, mice with reduced BM-PDGFRα^+^ mesenchymal cells showed massive inflammation of the grafts compared to mice that had normal levels of these cells even after HMGB1 administration, suggesting that BM-PDGFRα^+^ mesenchymal cells contribute to the HMGB1-induced anti-inflammatory effect. We also found that intravenously administered HMGB1 augmented the local migration of BM-PDGFRα^+^ mesenchymal cells from circulation to skin graft by inducing the expression of CXCR4, an SDF-1 receptor, on these cells. Finally, we showed the therapeutic activity of the HMGB1/BM-PDGFRα^+^ mesenchymal cell axis in an allergic contact dermatitis model. The results illustrated the contribution of the HMGB1/BM-PDGFRα^+^ mesenchymal cell axis in suppressing the inflammation of injured/inflamed skin. These findings may provide future perspectives on the use of HMGB1-based medicines for intractable diseases.

High-mobility group box 1 (HMGB1) is a non-histone nuclear protein that regulates chromatin structure remodeling as a molecular chaperone in the chromatin DNA-protein complex^1^. In injured/infected tissues, however, HMGB1 is actively secreted by macrophages and dendritic cells^2^,^3^ or passively released from necrotic cells^4^, and HMGB1 induces tissue remodeling by activating inflammatory reactions, i.e., macrophage and neutrophil infiltrations, via ligation to Toll-like receptors and/or the receptor for advanced glycation end-product on their surfaces^5^,^6^.

HMGB1 has also been reported to play a role in tissue regeneration. The local administration of HMGB1 was shown to promote tissue regeneration in myocardial infarction or diabetic ulcer by attenuating the inflammation or promotion of angiogenesis^7^,^8^. HMGB1 is also a strong chemoattractant for mesoangioblasts and endothelial precursor cells^9^,^10^. Despite these well-reported functions of locally injected HMGB1, it remains unclear whether systemic HMGB1 injection also promotes tissue regeneration.

Skin regeneration is a coordinated process with mutual interactions among various cell types, extracellular matrix and signaling molecules. Previous studies have indicated that well-regulated inflammatory reactions have positive impacts on the outcome of wound healing^11^. However, the wound-activated inflammatory reactions must be suppressed in the subsequent regeneration process, suggesting that a therapeutic strategy of modulating the inflammatory phase in the regenerative process might promote successful cutaneous wound repair.

Mesenchymal stromal cells (MSCs) in bone marrow (BM) are described as multi-potent cells with the ability to differentiate into osteocytes, adipocytes, and chondrocytes *in vitro*^12^,^13^. In addition to their multi-differentiation potentials, MSCs also possess potent anti-inflammatory activities. Specifically, MSCs secrete the tumor necrosis factor-alpha (TNF-α)-stimulated gene 6 protein (TSG-6), which attenuates the activation of resident macrophages^14^. MSCs have also been shown to activate interleukin-10 (IL-10) production from M2 macrophages, which suppresses inflammatory reactions by inhibiting both neutrophil infiltration and T-cell responses^15^,^16^.

The above-described characteristics of cultured MSCs have prompted the clinical use of these cells for the repair of various types of tissue damage, including skin wounds, in the field of regenerative medicine. Over the last decades, increasing evidence has indicated that the administration of cultured MSCs^17^,^18^ or their conditioned medium^19^,^20^ accelerates tissue regeneration. In cases of acute tissue injuries such as brain stroke^21^, myocardial infarction^21^ and bone fracture^22^, intravenously administered MSCs have been shown to specifically migrate to the injury sites by the action between stromal-derived factor-1α (SDF-1α) released from the injured tissues and its receptor CXCR4 expressed on the MSCs^23^.

To date, however, there have been few reports studying the behavior of the “endogenous” BM-MSCs during the tissue regeneration process. It was recently reported that the number of endogenous MSCs in BM decreased during experimental allergic encephalomyelitis, an animal model of multiple sclerosis^24^. Additionally, Hong et al reported that in a rabbit eye model of alkali burn, substance P, an injury-inducible factor, increased the number of stromal-like cells in circulation and enhanced wound healing^25^. These findings suggest that the activation of endogenous MSCs could be used to suppress inflammatory reactions and promote the regeneration process of damaged tissues.

We previously reported that BM-derived platelet-derived growth factor receptor-alpha (PDGFRα)^+^ mesenchymal cells accumulated in skin grafts^26^. In that model, the HMGB1 level was increased in serum after skin grafting. In addition, systemic recombinant HMGB1 administration mobilised endogenous PDGFRα^+^ mesenchymal cells from BM into circulation in the absence of damage, suggesting that HMGB1 could be used to stimulate endogenous PDGFRα^+^ mesenchymal cells for tissue regeneration. However, it is still unclear whether HMGB1-induced PDGFRα^+^ mesenchymal cells in circulation migrate to injury/inflammatory sites and promote the regeneration process.

In the present study, we investigated whether BM-PDGFRα^+^ mesenchymal cells mobilised from BM by intravenous HMGB1 administration would suppress the inflammatory reactions in skin grafts. We then investigated the mechanism by which HMGB1 regulates the SDF-1α/CXCR4 axis to achieve the accumulation of PDGFRα^+^ cells specifically in the wounded skin. Lastly, we evaluated the therapeutic effect of intravenous HMGB1 administration in a mouse model of allergic contact dermatitis (ACD), one of the most common inflammatory skin diseases.

## Results

### HMGB1 administration increased the accumulation of bone marrow-derived PDGFRα^+^ mesenchymal cells in skin grafts and suppressed inflammation

**Figure 1:**
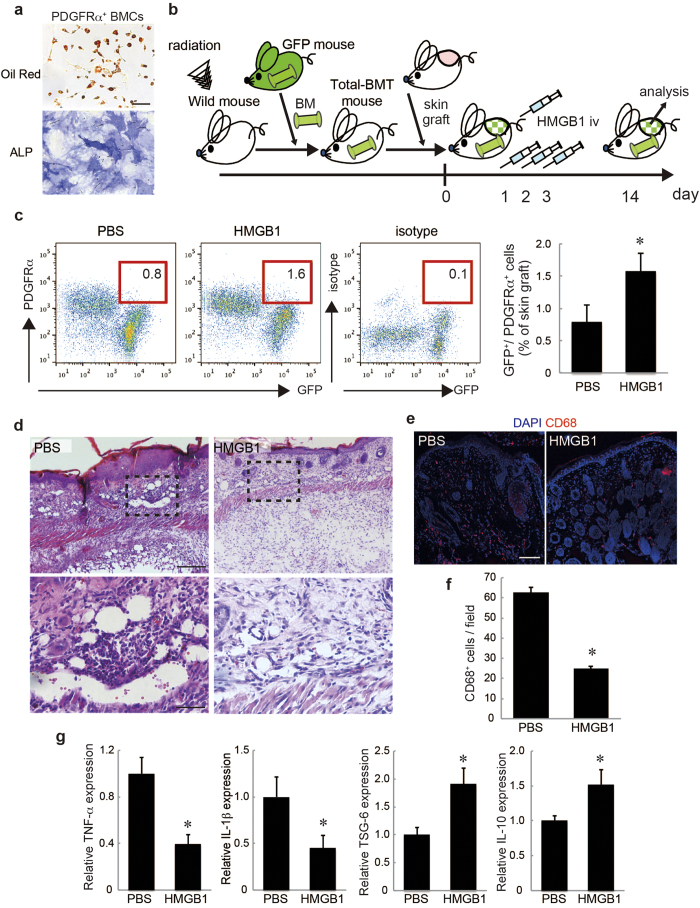
HMGB1 increased the accumulation of bone marrow-derived PDGFRα^+^ mesenchymal cells and suppressed inflammation in skin grafts. **a**: Oil-red staining and ALP staining. Bar = 100 μm. **b**: Schematic illustration of the skin graft model on GFP-bone marrow transplantation (BMT) mice. The picture was drawn by K.T. with Adobe illustrator CS6. **c**: The percentages of GFP^+^/PDGFRα^+^ cells recruited into skin grafts. **d**: Skin grafts stained by hematoxylin and eosin (H&E). The boxed regions in the upper panels (×10, bar = 200 μm) are displayed at higher magnification (×40, bar = 50 μm) in the lower panels. **e**: Representative images of skin grafts stained for CD68. Nuclei were stained with DAPI. Bar = 100 μm. **f**: The number of CD68^+^ cells per field was quantified. **g**: The expressions of TNF-α, IL-1β, TSG-6, and IL-10 in skin grafts. *P < 0.05.

To analyze the migration and function of endogenous BM-derived MSCs in skin, we first examined whether PDGFRα, which is known to be expressed in MSCs, could be a marker for BM-derived MSCs. PDGFRα^+^ cells isolated from BM had the ability to differentiate into both adipocytes and osteocytes (Fig. 1a), indicating that PDGFRα^+^ cells in BM have the potential to differentiate into multiple mesenchymal lineages. For our analysis of the accumulation of BM-derived PDGFRα^+^ cells in skin grafts after HMGB1 administration, green fluorescent protein-bone marrow transplantation (GFP-BMT) mice first received a skin graft from a wild-type mouse followed by intravenous HMGB1 administration (Fig. 1b). No BM-derived colony-forming cells were observed in the colony-forming unit (CFU) assay after irradiation (Suppl. Fig. S1a).

In addition to our previous report that almost all of the adherent BMCs derived from GFP-BMT mice were GFP^+^ cells^27^, we confirmed that GFP-BMT mice rarely had host-derived (GFP^–^) PDGFRα^+^ cells (Fig. S1b). These data suggest that the majority of the PDGFRα^+^ mesenchymal cells in BM were replaced with GFP^+^ cells in this GFP-BMT model. GFP^–^ cells and GFP^+^/PDGFRα^–^ cells isolated from the skin grafts did not adhere to a plastic dish (Fig. S2a), but GFP^+^/PDGFRα^+^ cells could adhere, and they also showed the ability to differentiate into both adipocytes and osteocytes (Fig. S2b).

In addition, the GFP^+^/PDGFRα^+^ cells specifically expressed some MSC markers such as PDGFRα and CD106^28^,^29^,^30^, suggesting that the population includes MSCs (Fig. S2c). Accordingly, we chose to analyze GFP^+^/PDGFRα^+^ cells as a population including BM-derived MSCs in the present study. By systemic HMGB1 administration, we found that a significant number of GFP^+^/PDGFRα^+^ cells were accumulated in the skin grafts at day 14 (Fig. 1c). Of note, a histological analysis of the skin grafts with or without HMGB1 administration showed that there was less infiltration of mononuclear cells in the HMGB1-administered group compared to its phosphate-buffered saline (PBS)-administered counterpart (Fig. 1d). Moreover, immunostaining for CD68, a marker for macrophages, showed that the skin grafts of HMGB1-treated mice exhibited a decrease in CD68^+^ cells (Fig. 1e,f).

Gene expressions of inflammation mediators such as TNF-α and IL-1β were significantly decreased in the skin grafts of the HMGB1-administered group (Fig. 1g). In contrast, TSG-6 and IL-10, which have been shown to be released from MSCs to suppress inflammation, were highly expressed in the grafts of the HMGB1-administered group (Fig. 1g). These results suggest that systemic HMGB1 administration augments the accumulation of BM-derived MSCs and suppresses the tissue inflammatory reactions, thereby promoting the tissue regeneration process of the skin graft.

### The reduction of PDGFRα^+^ mesenchymal cells in bone marrow exacerbated the skin graft regeneration

To determine whether the suppression of inflammatory reactions in the skin grafts depends primarily on the accumulation of the BM-derived PDGFRα^+^ cells, we prepared PDGFRα^+^ cell-reduced BMT mice to evaluate the inflammatory reactions in comparison with whole BMT mice. First, lineage-negative (Lin^−^)/ckit^+^ bone marrow cells (BMCs), which include hematopoietic stem cells, were confirmed not to include any colony-forming cells in a CFU assay (Fig. S3a) and not to express MSC markers such as PDGFRα and CD106 (Fig. S3b). These results suggest that Lin^−^/ckit^+^ BMCs rarely include PDGFRα^+^ mesenchymal cells.

**Figure 2:**
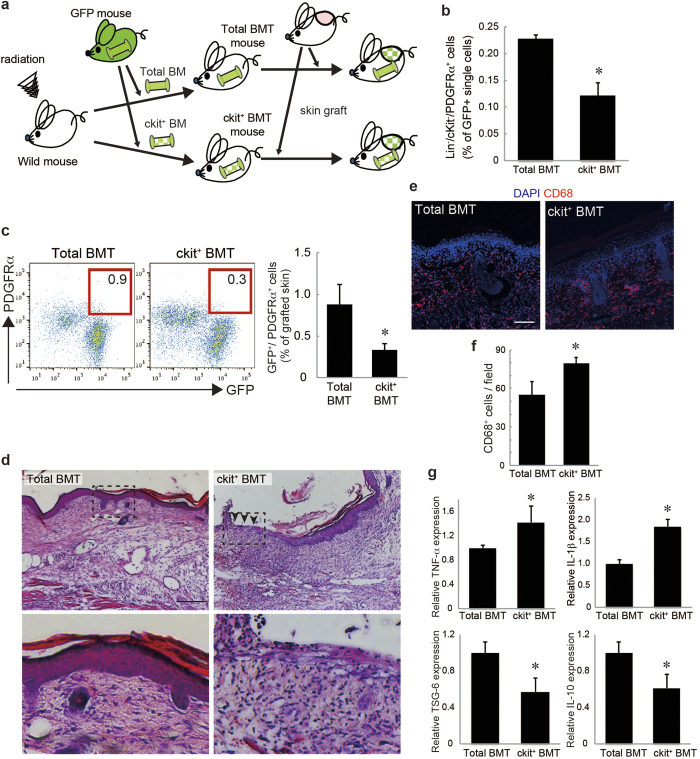
The reduction of endogenous PDGFRα^+^ mesenchymal cells delayed skin graft regeneration. **a**: The skin graft model in ckit^+^ BMT mice. The picture was drawn by K.T. with Adobe illustrator CS6. **b**: The percentages of Lin^−^/ckit^−^/PDGFRα^+^ cells among GFP^+^ single cells in the BM of the ckit^+^ BMT mice. **c**: The percentages of GFP^+^/PDGFRα^+^ cells recruited into skin grafts on the total and ckit^+^ BMT mice. **d**: Skin grafts stained by H&E. The boxed regions in the upper panels (×10, bar = 200 μm) are displayed at higher magnification (×40, bar = 50 μm) in the lower panels. **e**: Representative images of skin grafts stained for CD68. Nuclei were stained with DAPI. Bar = 100 μm. **f**: The number of CD68^+^ cells per field was quantified. **g**: The expressions of TNF-α, IL-1β, TSG-6, and IL-10 in skin grafts. *P < 0.05.

Next, this population was transplanted (ckit^+^ BMT) instead of total BMCs (total BMT) in mice after lethal-dose irradiation, and inflammatory changes of the skin grafts on the ckit^+^ BMT mice were compared with the skin grafts on the total BMT mice (Fig. 2a). Before the skin grafting, we confirmed that the ckit^+^ BMT mice had no colony-forming cells by CFU assay (Fig. S3c) and significantly less PDGFRα^+^ mesenchymal cells in BM compared to the total BMT mice by a flow cytometry analysis (Fig. 2b). The accumulation of BM-derived PDGFRα^+^ cells in the skin grafts on the ckit^+^ BMT mice was significantly reduced compared to that on the total BMT mice (Fig. 2c).

Histological observation of the skin grafts on the ckit^+^ BMT mice demonstrated higher inflammation, as suggested by increased infiltration of mononuclear cells and extravasation of erythrocytes, and loss of skin integrity, possibly due to the necrotic changes (arrowheads in Fig. 2d). Consistent with these results, the skin grafts of the ckit^+^ BMT mice had increased levels of CD68^+^ cells (Fig. 2e,f). In addition, the expressions of TNF-α and IL-1β were significantly increased in the skin grafts on the ckit^+^ BMT mice (Fig. 2g). On the other hand, the expressions of TSG-6 and IL-10 were significantly decreased in the skin grafts on ckit^+^ BMT mice (Fig. 2g). These data suggest that the decreased accumulation of BM-PDGFRα^+^ cells in grafted skin exacerbated the inflammatory reactions and necrotic changes in the graft.

### The anti-inflammatory effect of HMGB1 administration was abolished by the reduction of PDGFRα^+^ mesenchymal cells in bone marrow

**Figure 3:**
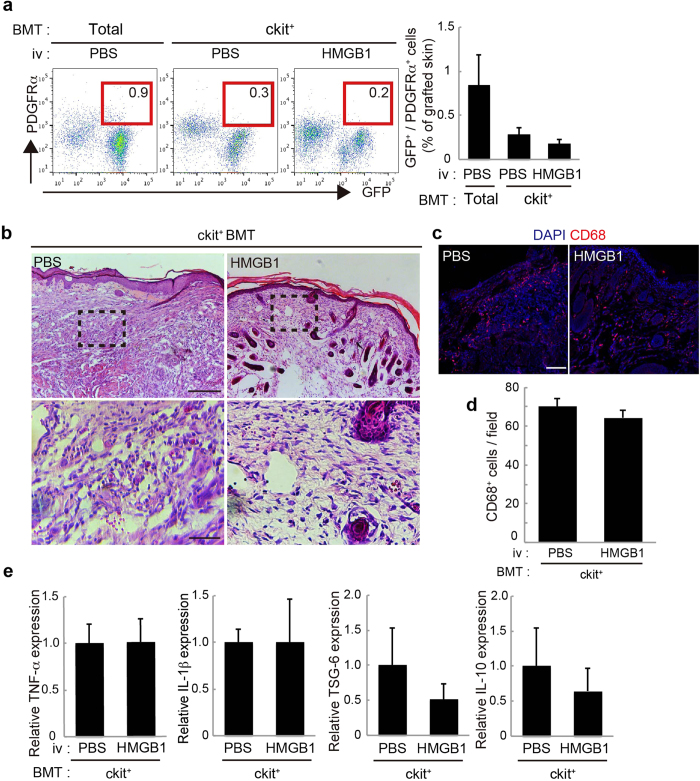
The anti-inflammatory effect of HMGB1 was canceled in the absence of PDGFRα^+^ mesenchymal cells. **a**: The percentages of GFP^+^/PDGFRα^+^ cells recruited into skin grafts on PBS-administered total BMT mice, and PBS- or HMGB1-administered ckit^+^ BMT mice. **b**: Skin grafts stained by H&E. The boxed regions in the upper panels (×10, bar = 200 μm) are displayed at higher magnification (×40, bar = 50 μm) in the lower panels. (**c**): Representative images of skin grafts stained for CD68. Nuclei were stained with DAPI. Bar = 100 μm. (**d**): The number of CD68^+^ cells per field was quantified. **e**: The expressions of TNF-α, IL-1β, TSG-6, and IL-10 in skin grafts (n = 4–5). *P < 0.05.

To confirm whether the anti-inflammatory effect of HMGB1 administration was exerted by the BM-derived PDGFRα^+^ cells accumulated in the graft, we next administered HMGB1 to the ckit^+^ BMT mice with skin grafts. In these mice, systemic HMGB1 administration did not augment the accumulation of PDGFRα^+^ cells in the skin grafts (Fig. 3a). Consistent with this result, no significant differences were observed in histological inflammatory changes or in the infiltration of CD68^+^ cells in the skin grafts between PBS- and HMGB1-administered ckit^+^ BMT mice (Fig. 3b–d). The expressions of inflammatory mediators (TNF-α, IL-1β) and anti-inflammatory mediators (TSG-6, IL-10) in the skin grafts were also not changed by HMGB1 injection (Fig. 3e). These results suggest that the anti-inflammatory reactions induced by HMGB1 administration depend mainly on the augmentation of endogenous BM-PDGFRα^+^ cell accumulation in the skin grafts.

### HMGB1 accelerated the accumulation of BM-derived PDGFRα^+^ mesenchymal cells in skin grafts by inducing CXCR4 expression on PDGFRα^+^ mesenchymal cells

**Figure 4:**
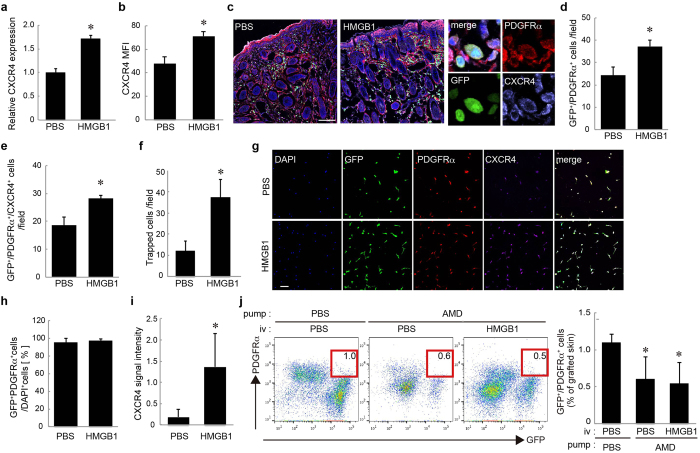
HMGB1 augmented the recruitment of PDGFRα^+^ mesenchymal cells by CXCR4 upregulation. **a**: The expressions of CXCR4 in cultured MSCs stimulated with or without HMGB1. **b**: Flow cytometry analysis for CXCR4 on BM-PDGFRα^+^ mesenchymal cells after systemic HMGB1 administration. **c**: Representative images of skin grafts stained for PDGFRα (red) and CXCR4 (purple). **d, e**: The numbers of GFP^+^/PDGFRα^+^ cells (d) and GFP^+^/PDGFRα^+^/CXCR4^+^ cells (e) per field were quantified. **f**: The number of TECs after a systemic administration of HMGB1 or PBS. **g**: Tube-entrapped cells (TECs) stained for PDGFRα (red) and CXCR4 (purple). **h**: The proportion of GFP^+^/PDGFRα^+^ cells in TECs. **i**: The signal intensity of immunostaining for CXCR4. **j**: Skin grafts on mice systemically antagonised with CXCR4. The percentages of GFP^+^/PDGFRα^+^ recruited into skin grafts with or without HMGB1 administration are shown. Nuclei were stained with DAPI. Bars = 100 μm. *P < 0.05.

We previously reported that HMGB1 mobilises BM-PDGFRα^+^ cells into circulation^26^. However, it has been unclear whether HMGB1 also affects the process of the migration of PDGFRα^+^ cells from the circulation to the injury sites. As the SDF-1α/CXCR4 axis generally plays a key role in the local migration of BM-MSCs, we assessed the CXCR4 expression on BM-PDGFRα^+^ cells after stimulation with HMGB1. We found that the CXCR4 mRNA expression was significantly elevated after 24 h of incubation with HMGB1 in BM-derived primary MSCs in culture (Fig. 4a). A flow cytometry analysis revealed that the CXCR4 protein level was also increased on the MSCs in the BM of the HMGB1-administered mice (Fig. 4b).

An immunostaining analysis demonstrated that HMGB1 administration increased not only GFP^+^/PDGFRα^+^ cells (Fig. 4c,d) but also GFP^+^/PDGFRα^+^/CXCR4^+^ cells in the skin grafts (Fig. 4e). The SDF-1α expression was increased in the skin grafts, but the expression level was not additionally augmented by HMGB1 administration (Fig. S4a,b). To further confirm whether the HMGB1-mediated CXCR4 expression level involves the accumulation of BM-PDGFRα^+^ cells at the SDF-1α-expressing sites, we subcutaneously implanted silicon tubes containing SDF-1α protein in the GFP-BMT mice to evaluate the accumulation of BM-PDGFRα^+^ cells with or without HMGB1 administration. After 2 days, we recovered the implanted tubes and cultured the tube-entrapped cells (TECs).

We found that HMGB1 injection indeed augmented the accumulation of BM-derived GFP^+^ cells in SDF-1α-containing tubes (Fig. 4f). Almost all of the adhered TECs in culture were BM-derived and PDGFRα^+^ cells (Fig. 4g,h). Additionally, the CXCR4 fluorescence intensity on the TECs was relatively higher in the HMGB1-administered group than the control PBS group (Fig. 4g,i), suggesting that BM-PDGFRα^+^ cells with higher CXCR4 expression induced by HMGB1 efficiently migrate to the injured/inflamed region with higher SDF-1α expression.

To test whether the BM-PDGFRα^+^ cells’ migration by HMGB1 was regulated mainly by the SDF-1α/CXCR4 axis, we analysed the HMGB1-induced accumulation of BM-PDGFRα^+^ cells in the skin grafts with or without systemic CXCR4-specific antagonist AMD3100 treatment. We found that the accumulation of BM-PDGFRα^+^ mesenchymal cells in the skin grafts was significantly decreased compared to that in the PBS-treated mice (Fig. 4j). Moreover, the HMGB1-induced augmentation of the BM-PDGFRα^+^ cells’ accumulation was clearly diminished by AMD3100 (Fig. 4j). These data suggest a correlation between the upregulation of CXCR4 on PDGFRα^+^ cells by HMGB1 and the accumulation of the BM-derived PDGFRα^+^ cells in the skin graft.

### Systemic HMGB1 administration ameliorated the inflammatory changes in allergic contact dermatitis

**Figure 5:**
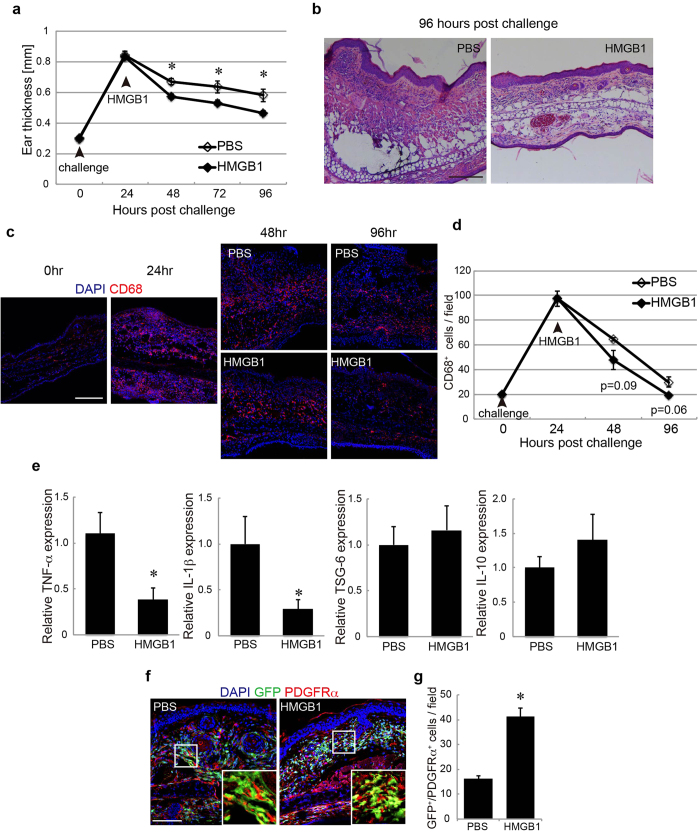
HMGB1 induced changes in the ear skin of ACD model mice. **a**: Ear thickness changes following PBS (white) or HMGB1 (black) administration. **b**: H&E staining of ear skin. Bar = 100 μm. **c**: Ear skin regions stained with CD68 in GFP-BMT mice at each time point after the challenge. **d**: The number of CD68^+^ cells per field was quantified at each time point. **e**: The expressions of TNF-α, IL-1β, TSG-6, and IL-10 in ear skin. **f**: Ear skin regions stained for PDGFRα in GFP-BMT mice at 96 h after the challenge. **g**: The number of GFP^+^/PDGFRα^+^ cells per field was quantified. **h**: Ear skin regions stained for SDF-1α in the GFP-BMT mice at 96 h after the challenge. Nuclei were stained with DAPI. Bar = 100 μm. *P < 0.05.

As the final investigation, the therapeutic anti-inflammatory activity of the HMGB1/PDGFRα^+^ mesenchymal cells axis was assessed in the mouse model of ACD. Mice were sensitised by an application of oxazolone (oxz) to the abdominal skin, followed by a single challenge of oxz on the ear skin 5 days later. The topical oxz application caused a typical hypersensitivity response, resulting in dermal edema formation with a more than twofold increase in thickness 24 h after the challenge (Fig. 5a). Systemic HMGB1 administration after the challenge significantly accelerated the resolution of the edema as evaluated by ear thickness (Fig. 5a). The infiltration of mononuclear cells was also decreased by the HMGB1 administration (Fig. 5b). The infiltration of CD68^+^ macrophages was increased 24 h after the challenge and then gradually decreased with time (Fig. 5c,d). In addition, this reduction began earlier in the HMGB1-administered mice than in their PBS-administered counterparts (Fig. 5c,d).

These histological data indicate that HMGB1 administration accelerates the resolution of inflammation. In agreement with the histological improvement, the gene expressions of TNF-α and IL-1β were decreased by HMGB1 administration (Fig. 5e). In contrast, the expressions of TSG-6 and IL-10 were not significantly changed (Fig. 5e). These data indicate that the systemic HMGB1 administration significantly suppressed the inflammatory changes of the skin with ACD.

For the investigation of whether HMGB1 would augment the migration of BM-MSCs to the inflammatory skin in the ACD model mice, GFP-BMT mice were challenged with oxz to induce ACD. The number of BM-derived PDGFRα^+^ cells (GFP^+^/PDGFRα^+^ cells) in the ear skin of the HMGB1-administered GFP-BMT mice was increased compared to that in the controls (Fig. 5f,g). This result suggests a correlation between the anti-inflammatory effect of HMGB1 administration and BM-PDGFRα^+^ cells accumulation in the ACD lesions.

## Discussion

The aim of this study was to examine whether mobilising endogenous PDGFRα^+^ mesenchymal cells with a systemic injection of HMGB1 could be a therapeutic strategy for tissue regeneration. The effectiveness of transplanting culture-expanded MSCs is currently being investigated in clinical trials for various tissue repair and immunological disorders. However, the preparation of cultured MSCs is fairly labor-intensive and requires specialized culture equipment for their expansion. In contrast, the mobilisation of specific endogenous MSC populations does not require extensive *ex vivo* manipulation or vehicles for delivery. In addition, there is growing evidence that culture-expanded MSCs lose both their damage site-homing ability and their anti-inflammatory functions during the expansion period in culture^31^,^32^,^33^,^34^. We therefore believe that the endogenous MSC recruiting strategy not only skips the process necessary for *ex vivo* expansion, but may also induce MSCs with more therapeutic potency than culture-expanded MSCs.

HMGB1 is well known to have multi-functional cytokine activities when released into the extracellular milieu in addition to its chromatin remodeling actions in the nuclei. HMGB1 forms heterocomplexes with other cellular or bacterial molecules, such as DNA, RNA, histones, or lipopolysaccharide (LPS), to generate synergistic innate immune responses stronger than those of the individual components^35^,^36^. In injured/infected tissues, these HMGB1-heterocomplexes bind to Toll-like receptors (TLRs) on the dendritic cells and macrophages, which then release chemoattractants and proinflammatory cytokines, resulting in acute and chronic inflammation^5^,^6^.

In addition to these functions, we previously found that the free form of systemically injected HMGB1 mobilizes the endogenous BM-PDGFRα^+^ mesenchymal cells into circulation^26^. Therefore, in the present study, we investigated whether a systemic injection of free-form HMGB1 and the resulting mobilization of endogenous BM-PDGFRα^+^ mesenchymal cells could be used as a therapeutic strategy in skin injury models. The results first demonstrated the possibility that the accumulation of endogenous BM-PDGFRα^+^ mesenchymal cells might be correlated with the observed improvement of inflammatory change in the skin grafts.

Our analysis of BM-derived PDGFRα^+^ mesenchymal cells relied on the GFP-BMT model. We confirmed that the majority of the PDGFRα^+^ mesenchymal cells in BM were replaced with GFP^+^ cells in this GFP-BMT model. However, we cannot exclude the possibility of a contribution by radiation-resistant host BM-MSCs. Although further studies are required to confirm a direct relationship between the accumulation of PDGFRα^+^ mesenchymal cells and skin pathology, the results obtained here with HMGB1-administered mice and those for the ckit^+^ BMT mice suggest a correlation between the accumulation of BM-PDGFRα^+^ mesenchymal cells and inflammatory changes.

In addition, the results of the HMGB1 administration to ckit^+^ BMT mice further support our hypothesis that a sufficient number of endogenous PDGFRα^+^ mesenchymal cells in BM was required to achieve a therapeutic effect by HMGB1 administration. However, since HMGB1 has multiple functions in various cell types, it should be determined whether the extraneous transplantation of freshly isolated BM-PDGFRα^+^ mesenchymal cells into damaged skin enhances skin regeneration in a ckit^+^ BMT model. Moreover, as we analyzed the accumulation of PDGFRα^+^ mesenchymal cells in skin grafts using flow cytometry, the absolute number of these cells was not confirmed. Further studies will be needed to confirm whether HMGB1 injection causes the increase of the absolute number of PDGFRα^+^ cells or changes the population in skin cells.

In addition to the variety of HMGB1 targets, several reports have suggested that PDGFRα^+^ mesenchymal cells contain sub-populations with distinct functions, such as the maintenance of HSC niches and immunosuppressive activities^28^,^37^,^38^. In the present study, we focused on PDGFRα^+^ cells in BM as a population including MSCs. Our finding that only GFP^+^/PDGFRα^+^ cells from skin grafts adhered to a plastic dish and expressed some MSC markers suggests that this population includes BM-derived MSCs. However, we found some cells in the PDGFRα^+^ cell population which have no capacity to differentiate into adipocytes (Fig. 1a, Fig. S2b). These results suggest that both the BM-PDGFRα^+^ population and the GFP^+^/PDGFRα^+^ population in skin grafts are highly heterogeneous. Given that HMGB1 also induce other cell types^9^, it will thus be important to further identify specific PDGFRα^+^ subpopulations with immunosuppressive functions that are induced by HMGB1.

MSCs have several well-known inflammatory actions, such as the secretion of various cytokines (including TSG-6 and IL-10) to suppress inflammatory reactions^39^. Indeed, in the present experiments TSG-6 expression was significantly elevated in the skin grafts when large amounts of PDGFRα^+^ mesenchymal cells were accumulated there in response to HMGB1. In the ACD model, however, HMGB1 enhanced the PDGFRα^+^ mesenchymal cells’ accumulation and suppressed the expression of pro-inflammatory mediators such as TNF-α and IL-1β without inducing TSG-6 or IL-10 expression. These data suggest that there are other anti-inflammatory mechanisms of PDGFRα^+^ mesenchymal cells in ACD lesions.

We previously reported that the serum HMGB1 level was increased 3 days after skin grafting in mice^26^, indicating the release of endogenous HMGB1 in the skin grafts. In the ACD mice, however, the HMGB1 level in sera was not increased (data not shown), indicating that the level of HMGB1 released in ACD skin was lower than that in the skin grafts. These pathological differences—including differences in the HMGB1 level—between the ACD and skin graft model may have been involved in the different anti-inflammatory actions of PDGFRα^+^ mesenchymal cells in each model.

Previously, we and others demonstrated that BM-MSCs or PDGFRα^+^ mesenchymal cells migrate to damage sites via the CXCR4/SDF-1α axis^22^. Previous studies on the transplantation of *ex vivo* expanded MSCs demonstrated that CXCR4 upregulation enhanced both the migration of MSCs and their therapeutic effects^21^,^31^. When considered together with our present finding that HMGB1 induced CXCR4 upregulation, these results suggest that HMGB1 may enhance the migration of PDGFRα^+^ mesenchymal cells to skin lesions expressing SDF-1α by upregulating CXCR4 expression. However, as PDGFRα^+^/CXCR4^−^ cells were also observed in skin, there is still the possibility that an axis other than CXCR4/SDF-1α makes some contribution to the PDGFRα^+^ mesenchymal cells’ recruitment. Further studies will be needed to confirm whether the upregulation of CXCR4 by HMGB1 directly enhances the accumulation of BM-PDGFRα^+^ mesenchymal cells in damaged skin.

Though there is still no direct evidence of a correlation between the anti-inflammatory effects of HMGB1 and the migration of PDGFRα^+^ mesenchymal cells, we here demonstrated that HMGB1 administration enhanced the accumulation of PDGFRα^+^ mesenchymal cells and suppressed inflammation in skin lesions. Our findings, together with previous reports about the pro-inflammatory aspects of HMGB1, may raise questions about the apparent bimodal function of HMGB1, since this protein appears to both activate inflammation in the initial phase of injured/infected tissues and subsequently inactivate inflammation by inducing an accumulation of PDGFRα^+^ mesenchymal cells and promoting tissue regeneration. Further studies will be required to more precisely define these bimodal HMGB1 functions for maintaining tissue homeostasis.

In conclusion, we demonstrated that a systemic administration of HMGB1 accelerated the accumulation of endogenous BM-PDGFRα^+^ mesenchymal cells in the injured skin and suppressed cutaneous inflammation. The observations provided here may illustrate the contribution of the HMGB1/BM-PDGFRα^+^ mesenchymal cell axis in promoting the healing of injured/inflamed skin. Our studies may provide future perspectives on HMGB1-based medicine for intractable diseases characterised by severe inflammation and tissue damage.

## Methods

### Mice

All animals were handled in accordance with the approved guidelines of the Animal Committee of the Osaka University Graduate School of Medicine. The protocols were approved by the Animal Committee of the Osaka University Graduate School of Medicine. All experimental mice were housed in cages with 12-h light-dark cycles. Solid food and water were supplied *ad libitum*. C57BL/6 mice were purchased from CLEA Japan (Tokyo). Only male mice were used. C57BL/6 mice that ubiquitously express enhanced green fluorescent protein (GFP; the mice are referred to as GFP mice) were kindly provided by Masaru Okabe (Osaka University, Osaka, Japan).

### Isolation of bone marrow cells, bone marrow transplantation and culture of primary BM-MSCs

The isolation of BMCs and BMT was performed as described^22^,^26^. Briefly, under sterile conditions, BMCs were isolated by flushing the femurs and tibiae with 2% fetal bovine serum (FBS)/PBS, and the resulting suspension was then filtered through 40-μm cell strainers. The filtrate was centrifuged at 4 °C for 10 min at 1500 rpm, and the pellet was suspended in PBS. For the total BMT, 5 × 10^6^ BMCs were injected into the tail vein of mice irradiated with 10 Gy. Four to six weeks after reconstitution, the BMCs were obtained to examine the chimerisms by measuring GFP^+^ cells stained with APC-conjugated lineage (Lin) antibody (BD Pharmingen, Bedford, MA).

Fluorescence was measured by a BD FACSCanto™ II system (BD Pharmingen), and it was confirmed that 85%–90% of total BMCs expressed GFP (data not shown). For the ckit^+^ BMT model, the Lin^−^ BMCs from GFP mice isolated using a magnetic cell sorting (MACS) system (Miltenyi Biotec, Bergisch Gladbach, Germany) were stained with APC-conjugated c-kit antibody (clone 2B8; eBioscience, San Diego, CA). Then, Lin^−^/GFP^+^/ckit^+^ BMCs were sorted with a BD FACSAria™ II cell sorter (BD Pharmingen). Each irradiated mouse received 1 × 10^5^ – 2 × 10^5^ GFP^+^/Lin^−^/ckit^+^ BMCs, which was almost identical to the number of GFP^+^/Lin^−^/ckit^+^ BMCs used for the total BMT. After reconstitution, the numbers and percentages of donor derived-MSC populations in BM were examined (Fig. 2b). Isolated BMCs were cultured with α-MEM containing 20% FBS and antibiotics.

### Skin graft model

The skin grafts were performed as described^26^. Full-thickness skin from wild-type newborn mice (2 × 2 cm) was carefully isolated by excision after the mice had been euthanised under systemic anesthesia, and engrafted onto the backs of the GFP-BMT mice just above the muscular fascia. The wound sites on the skin-grafted mice were then covered with bandaging tape to protect the grafted skin from scratching until further examination after engraftment. For therapeutic intervention, recombinant HMGB1 (10 μg in 100 μL) or PBS (100 μL) was intravenously administered for 4 successive days after skin grafting.

### Colony-forming unit assay

Lin^−^ BMCs were collected to exclude all hematopoietic lineage cells from total BMCs by the MACS system (Miltenyi Biotec). Isolated BMCs were then seeded in a 6-well plate. The adherent cells were stained as described^40^.

### Adipocyte and osteocyte differentiation *in vitro*

To induce adipocyte or osteocyte differentiation, we incubated culture-expanded BM-MSCs at passage 3 with either adipogenic medium or osteogenic medium using a Mesenchymal Stem Cell Adipogenesis Kit or Mesenchymal Stem Cell Osteogenesis Kit (Chemicon International, Temecula, CA). Three or four days later, intracellular lipid droplets were stained with a Lipid Assay kit (Cosmo Bio, Tokyo) according to the manufacturer’s instructions. Alkaline phosphatase activity was visualised using an Alkaline Phosphatase (ALP) staining kit (Primary Cell Co., Hokkaido, Japan) according to the manufacturer’s instructions.

### Histology

Cross-sections (8-μm thickness) of skin were cut in a cryostat (Leica Microsystems, Wetzlar, Germany) for the immunostaining analysis of the morphological properties. Hematoxylin and eosin (H&E) staining was carried out to check the degree of damage and the regeneration process. Briefly, cross-sections were air-dried at room temperature and stained with Mayer’s hematoxylin (Wako, Osaka, Japan) and 1.0% eosin (Wako).

### *In vivo* migration assay

The GFP-BMT mice were subcutaneously implanted with SDF-1α (100 ng/mL; Peprotech, Rocky Hill, NJ)-containing silicon tubes 24 h after an intravenous administration of HMGB1 (10 μg in 100 μL) or PBS (100 μL). Two days later, the tubes were collected and washed three times with α-MEM supplemented with 20% FBS, and the aliquots were centrifuged for 10 min at 1500 rpm. The pellets were then cultured for 2 days and analysed by immunostaining

### Immunostaining

The immunostaining of skin cross-sections was performed as described^26^. The cross-sections (8-μm thickness) were incubated with rat monoclonal anti-CD68 antibody (1:200; Abcam, Cambridge, MA), rat monoclonal anti-CXCR4 (1:200; R&D Systems, Minneapolis, MN), goat polyclonal anti-PDGFRα (1:20; R&D), or rabbit polyclonal anti-SDF-1α (1:200; eBioscience) at 4 °C overnight. The TECs were fixed in 4% paraformaldehyde (PFA) for 15 min, then washed and immunostained with rat monoclonal anti-CXCR4 (1:200; R&D) and goat polyclonal anti-PDGFRα (1:20; R&D) at 4 °C overnight. These cells were then stained with a secondary antibody, Alexa Fluor 546-conjugated goat anti-rabbit IgG (1:400; Molecular Probes, Eugene, OR). Stained cross-sections and cells were visualised using a confocal laser microscope (model A1/C1; Nikon, Tokyo) using NIS-Elements AR 3.1 software, and the images were arranged with Adobe Photoshop software.

### Delivery of CXCR4 antagonist

To ensure sufficient levels of the antagonist throughout the experimental period, we used osmotic Alzet pumps (Alza Corp., Mountain View, CA) to deliver the CXCR4 antagonist AMD3100 (Sigma-Aldrich, St. Louis, MO) at a constant rate of 10 mg/kg/day. The Alzet pumps were loaded with AMD3100 or PBS and implanted subcutaneously 1 h before the skin graft implantation.

### Fluorescence-activated cell sorting (FACS) analysis and sorting of bone marrow cells

Total BMCs were isolated as described above. Isolated BMCs containing 2 × 10^6^ cells were suspended in 200 μL of staining buffer (2% FBS in PBS), followed by incubation with purified rat anti-mouse CD16/CD32 (Mouse BD Fc Block™; BD Pharmingen) for 10 min at 4 °C. The following fluorescence-conjugated antibodies were then applied and incubated for 30 min at 4 °C: anti-mouse CD140a (PDGFRα APC (clone APA5; BioLegend, San Diego, CA), anti-mouse Lineage Cocktail with Isotype control APC (BD Pharmingen), anti-mouse CD117 (c-kit) APC (clone 2B8; eBioscience), and anti-mouse CD184 (CXCR4) APC (clone 2B11; eBioscience).

As matching isotype controls, fluorescence-conjugated anti-mouse IgG antibodies obtained from either BioLegend or eBioscience were used in each experiment. Stained cells were then analysed on the BD FACSCanto II system. For the sorting experiment, a BD FACSAria II was used. The sorting gates were defined based on the isotype control staining. The FACS data were analysed using FlowJo software, version 6.3.3 (Tree Star, Ashland, OR).

### Skin digestion for the flow cytometric analysis

The harvested skin grafts were minced into small pieces and digested using 0.2% collagenase A (Roche, Mannheim, Germany) for 40 min at 37 °C with frequent triturating using a pipette. Following digestion, the cells were filtered using 40-μm nylon mesh strainers, followed by centrifugation at 1,500 rpm for 10 min. The pellets were then suspended in the staining buffer and stained with the specific antibodies described above.

### RNA extraction and real-time PCR

Total RNA was extracted from the skin graft, ear and BMCs using ISOGEN (Nippon Gene, Toyama, Japan) according to the manufacturer’s instructions. In each experiment, cDNA was synthesised from an equivalent quantity of total RNA (2 μg for skin grafts and ears and 500 ng for sorted BMCs) by reverse transcription using a High Capacity RNA-to-cDNA kit (Applied Biosystems, Foster City, CA). The real-time PCR was performed with SYBR PremixEX Taq (Takara Bio, Shiga, Japan) when using oligonucleotide primers. The oligonucleotide primers were designed using the Universal Probe Library Assay Design Center (Roche). The sequences of oligonucleotide primers used for the real-time PCR were as follows: glyceraldehyde 3-phosphate dehydrogenase (GAPDH), sense 5’-ACTCCCACTCTTCCACCTTC-3’ and antisense 5’-TCTTGCTCAGTGTCCTTGC-3’; TNF-α, sense 5’-GCTCCAGTGAATTCGGAAAG-3’ and antisense 5’-GATTATGGCTCAGGGTCCAA-3’; IL-1β, sense 5’- TTGACGGACCCCAAAAGAT-3’ and antisense 5’-GAAGCTGGATGCTCTCATCTG-3’; TSG-6, sense 5’-AGGCAGCCAGAAAAATTGG-3’ and antisense 5’-CACAATGGGGTATCCGACTC-3’; IL-10, sense 5’-CAAGGAGCATTTGAATTCCC-3’ and antisense 5’- GGCCTTGTAGACACCTTGGTC-3’; PDGFRα, sense 5’- GACGAGTGTCCTTCGCCAAAGTG-3’ and antisense 5’- CAAAATCCGACCAAGCACGAGG-3’; CD106, sense 5’-TCTTACCTGTGCGCTGTGAC-3’ and anti-sense 5’- ACTGGATCTTCAGGGAATGAGT-3’; angiopoietin-1, sense 5’- TTGTGATTCTGGTGATTGTGG-3’ and antisense 5’- CTTGTTTCGCTTTATTTTTGT-3’ (all purchased from Invitrogen, Carlsbad, CA). The quantitative data were obtained in triplicate within a single experiment on a 384-well plate based on the standard curve method using CFX manager software (BioRad, Hercules, CA). All data were normalised by the GAPDH level as an internal control. Data are expressed as the fold increase versus the control value.

### Oxazolone-induced allergic contact dermatitis model

To induce ACD, we dissolved oxazolone (oxz) in a mixture of acetone:olive oil (4:1). Mice were sensitised with 2% oxz solution applied to the shaved abdomen (150 μL). Five days later, mice were challenged with 1% oxz applied to the ear. Ear thickness was measured before the challenge and again after the challenge at the times indicated, using a Digimatic Messscharaube mit verstellbarer (Mitutoyo Corp., Kawasaki, Japan). For therapeutic intervention, recombinant HMGB1 (10 μg in 100 μL) or PBS (100 μL) was intravenously administered 24 h after challenge. Ears were harvested 96 h after the challenge and analysed.

### Statistical analysis and microscopy

Values are expressed as means ±    SEM. The data were examined using Student’s *t*-test. For all statistical tests, significance was accepted at P < 0.05.

## Additional Information

**How to cite this article**: Aikawa, E. *et al*. Systemic high-mobility group box 1 administration suppresses skin inflammation by inducing an accumulation of PDGFRα^+^ mesenchymal cells from bone marrow. *Sci. Rep*. **5**, 11008; doi: 10.1038/srep11008 (2015).

## Supplementary Material

Supplementary Information
