# Supplementary material for: Systemic high-mobility group box 1 administration suppresses skin inflammation by inducing an accumulation of PDGFRα+ mesenchymal cells from bone marrow
Source: Sci Rep. 2015 Jun 5;5:11008. doi: 10.1038/srep11008 (PMC4457135; doi:10.1038/srep11008)
Supplement: Supplementary Information [file srep11008-s1.pdf]

Supplementary Information for

Systemic high mobility group box 1 administration suppresses skin inflammation by inducing an accumulation of PDGFRα$^+$ mesenchymal cells from bone marrow

Eriko Aikawa[1], Ryo Fujita[2], Yasushi Kikuchi[1], Yasufumi Kaneda[2], and Katsuto Tamai[1*]

[1]Department of Stem Cell Therapy Science and [2]Division of Gene Therapy Science,

Graduate School of Medicine, Osaka University, Japan

* Corresponding author: tamai@gts.med.osaka-u.ac.jp

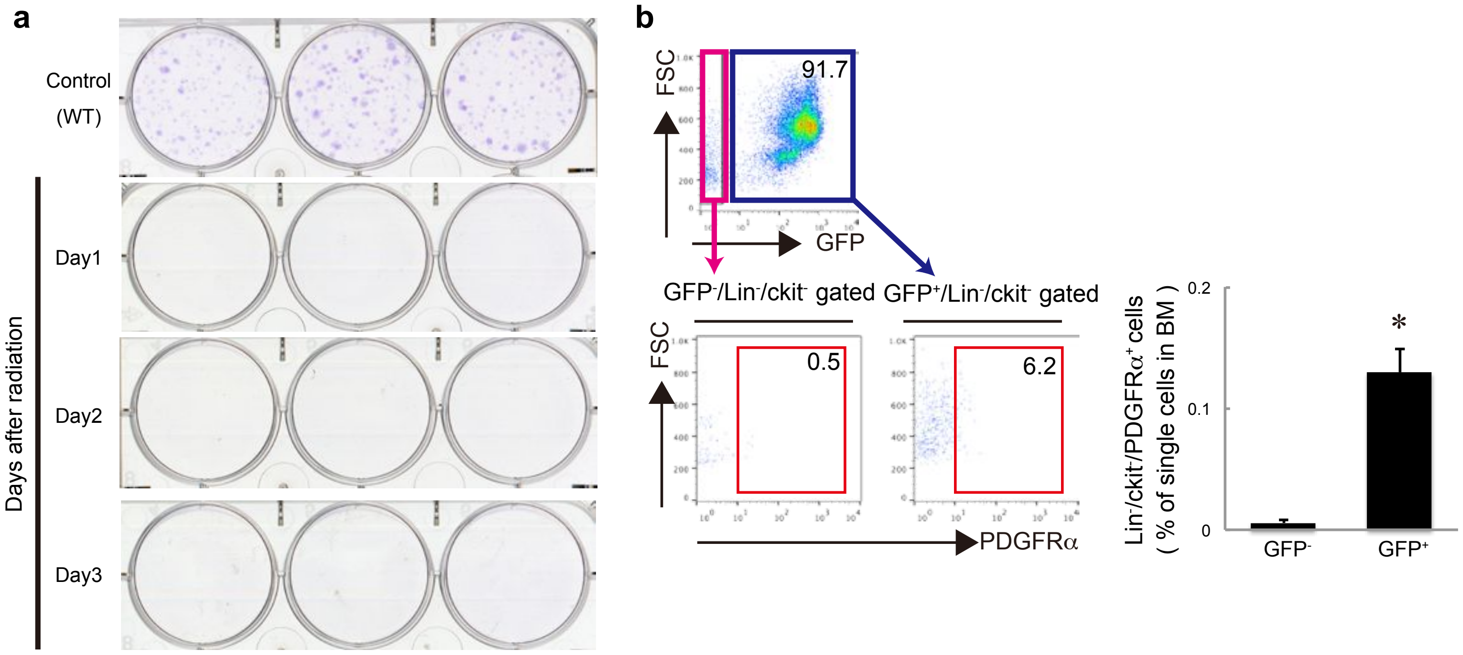

Figure S1. The GFP-BMT model was analyzed by CFU assay and flow cytometry. (a): Colony-forming unit (CFU) assay for bone marrow cells (BMCs) of wild type mice before and after irradiation. (b): Flow cytometry analysis of BMCs in GFP-BMT mice. The percentages of $Lin^-/ckit^-/PDGFR\alpha^+$ cells among GFP^+ or GFP^- cells are shown in the right panel.

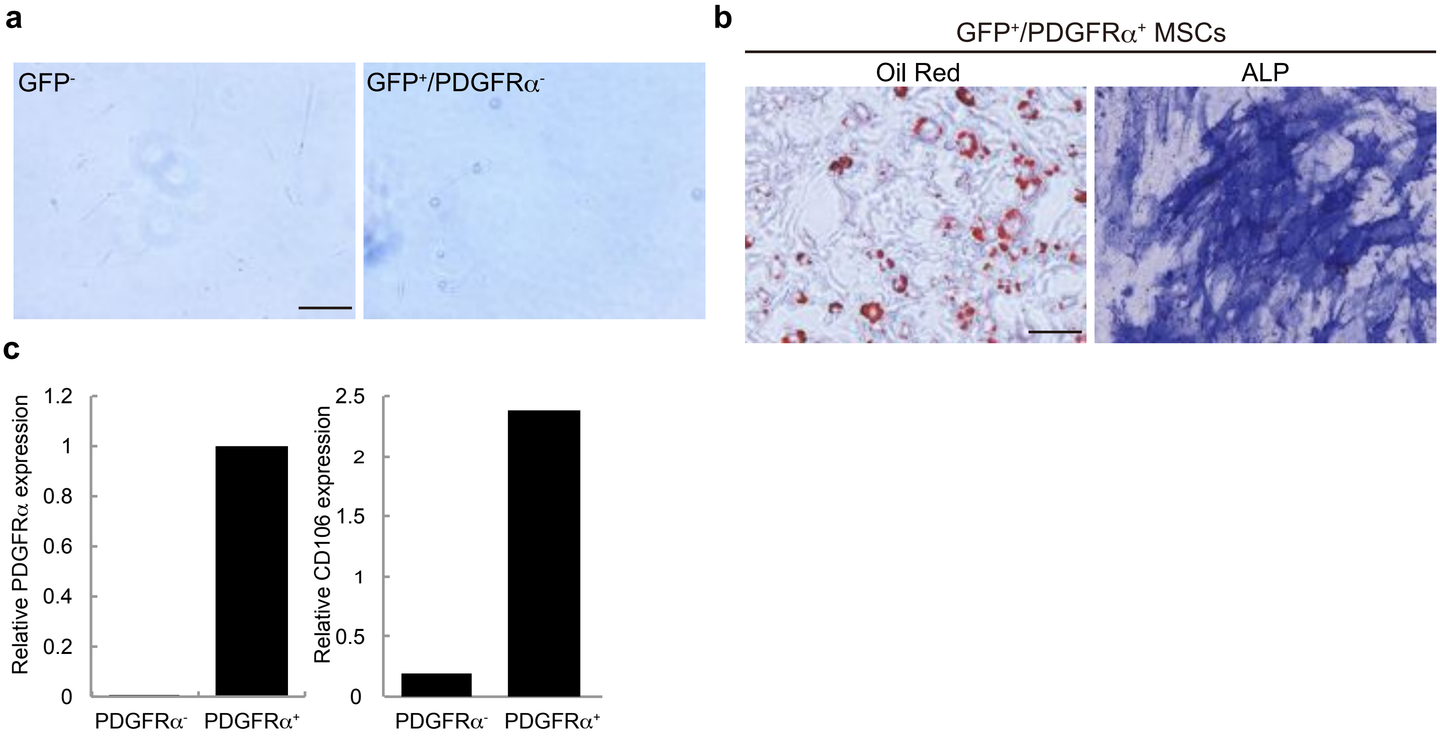

Figure S2. GFP$^+$/PDGFRα$^+$ cells in skin grafts have some characteristics of MSCs. (a): Representative pictures of GFP$^-$ cells and GFP$^+$/PDGFRα$^-$ cells isolated from skin grafts. (b): Oil-red staining and ALP staining. (c): The expressions of PDGFRα and CD106 in sorted cells from skin grafts. Bar = 200 μm.

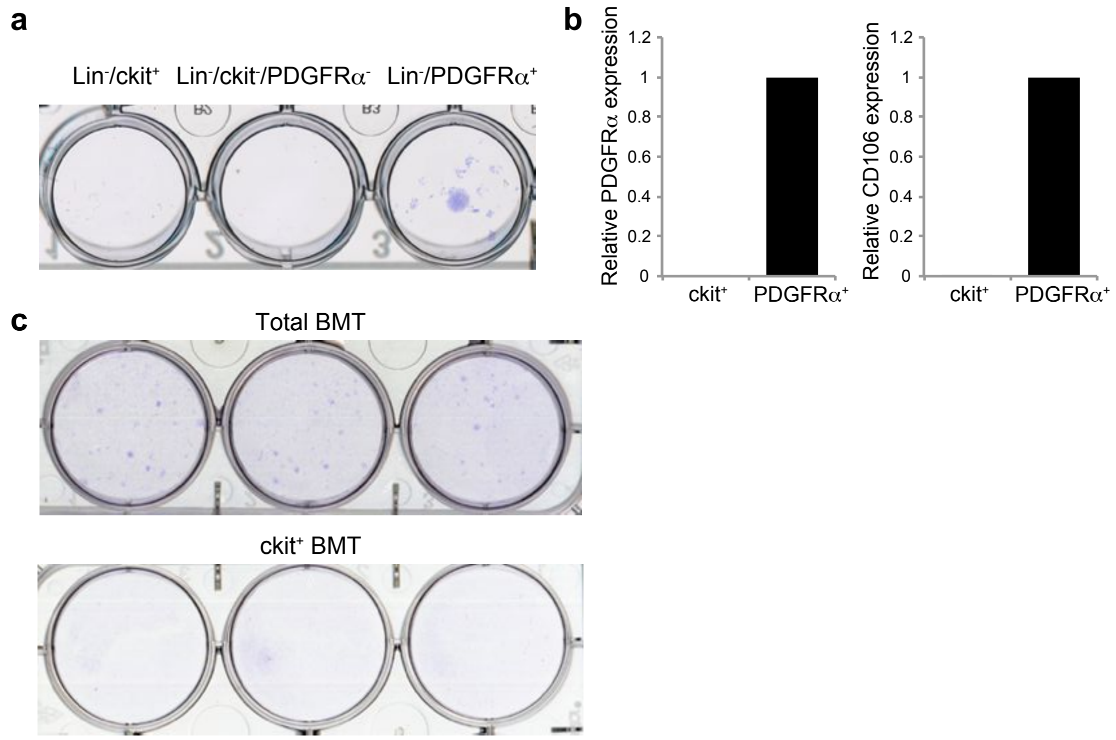

Figure S3. ckit$^+$ BM cells did not include PDGFRα+ mesenchymal cells. (a): CFU assay for freshly isolated BMCs. BMCs were FACS sorted to Lin$^-$/ckit$^+$ cells, Lin-/PDGFRα^+ cells, and Lin$^-$/ckit$^-$/PDGFRα^- cells. (b): The expressions of PDGFRα andCD106 in sorted BMCs. (c): Results of the CFU assay for BMCs BMT mice.

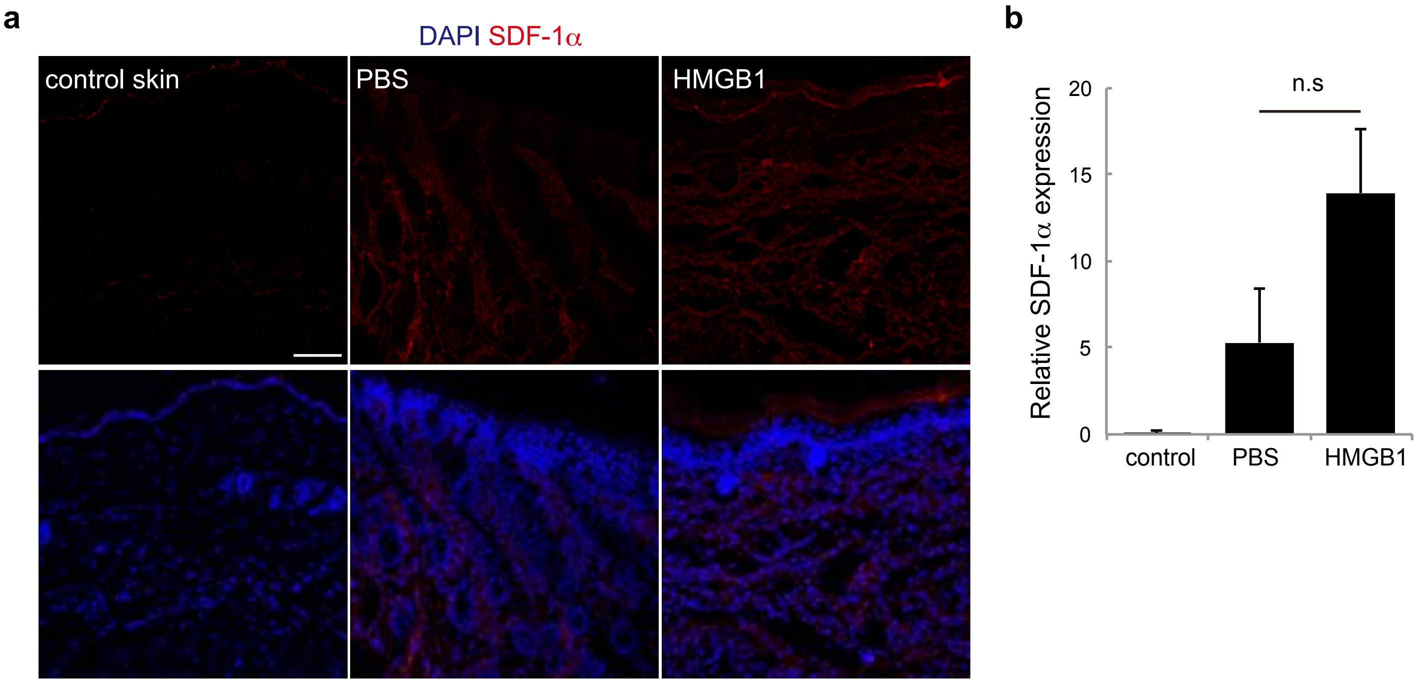

Figure S4. Skin grafts expressed SDF-1α. (a): Representative images of skin grafts stained for SDF-1α. Nuclei were stained with DAPI. Bar = 200 μm. (b): The expressions of SDF-1α in skin grafts with or without HMGB1 administration.
